# Blunt Aortic / Inferior Vena Cava Injury: Are We Consistently Providing the Same Level of Care?

**DOI:** 10.7759/cureus.6832

**Published:** 2020-01-31

**Authors:** Monica Leon, Luis O Chavez, Alda Chavez, Salim Surani

**Affiliations:** 1 General Surgery, ABC Medical Center, Mexico City, MEX; 2 Internal Medicine, Texas Tech University Health Sciences Center, El Paso, USA; 3 Faculty of Medicine and Psychology, Autonomous University of Baja California, Tijuana, MEX; 4 Internal Medicine, Texas A&M Health Science Center, Bryan, USA

**Keywords:** blunt trauma, aortic injury, inferior vena cava injury, vascular injury, shock, ivc

## Abstract

Major vascular traumatic injuries have a higher pre-hospital and in-hospital mortality rate. The different mechanisms of injury and anatomy of the aorta and inferior vena cava (IVC) make the management a constant challenge to surgeons and clinicians. Blunt traumatic aortic injury (BTAI) can occur at the thoracic or abdominal level, each of which possesses different considerations. Blunt traumatic inferior vena cava injury (BTIVCI) also has important diagnostic challenges since the lesion may not be as evident in the IVC as compared to the aorta, possibly due to lower caval pressures or the ability to self-tamponade from adjacent structures. Endovascular management has significantly increased in the past years, and despite an improvement in mortality, the approach to aortic and IVC injuries is not well standardized. Diagnostic imaging helps to classify the extent of the lesions and guide towards the best therapeutic options for each case. Conservative management, in some cases, has shown to reduce mortality, and close follow-up has proven good outcomes. Future research will provide more evidence to determine the best approach to BTAI and BTIVCI for better long-term outcomes. This article aims to provide an updated review of the current literature regarding diagnosis, classification, and management of BTAI and BTIVCI.

## Introduction and background

Trauma-related injuries are a leading cause of death worldwide that have increased over the last two decades [1]. Major vascular trauma such as injuries to the aorta or the inferior vena cava (IVC) has a high rate of pre-hospital mortality [2]. Blunt traumatic aortic injury (BTAI) is the second most common post-traumatic cause of death following traumatic brain injury as the first cause. According to the National Trauma Databank of the American College of Surgeons, from 2002-2014 the incidence of combined injuries to the IVC and the thoracic aorta was 1.1%, thoracic and abdominal aorta was 0.8% and IVC and abdominal aorta was 2.1% [1].

In the past, the standard of care of traumatic vascular injuries was open surgical repair, and the in-hospital mortality for BTAI in the first 24 hours was up to 50% of patients [3, 4]. However, in the last two decades, endovascular management for blunt trauma vascular injuries has significantly increased from 0.4% in 2002 to 13.2% in 2010. Parallel to these findings, the overall mortality from aortic and IVC blunt trauma decreased from 48.8% in 2002 to 28.7% in 2014, with a more significant reduction in the BTAI group [1]. The use of endovascular techniques has decreased morbidity and mortality from traumatic blunt vascular trauma [1, 5]. The aim of this article is to provide an updated literature review of the diagnosis, classification and current management of both BTAI and blunt traumatic inferior vena cava injury (BTIVCI).

## Review

Blunt traumatic aortic injury

Mechanism of injury in blunt trauma to aorta includes tearing of the aorta that is pinched between the vertebral body or displaced sternum, torsion and shearing forces, rapid deceleration and increased intravascular pressure. The most common site of injury is the aortic isthmus, followed by the ascending aorta and diaphragmatic hiatus. During the initial evaluation, findings suggestive of BTAI in a chest radiograph (CXR) include mediastinal widening (more than eight centimeters) at the aortic arch, irregularity of the aortic arch, loss of definition of the aortopulmonary window, deviation of the trachea, widening of left para-tracheal stripe, among others [3]. 

**Figure 1:**
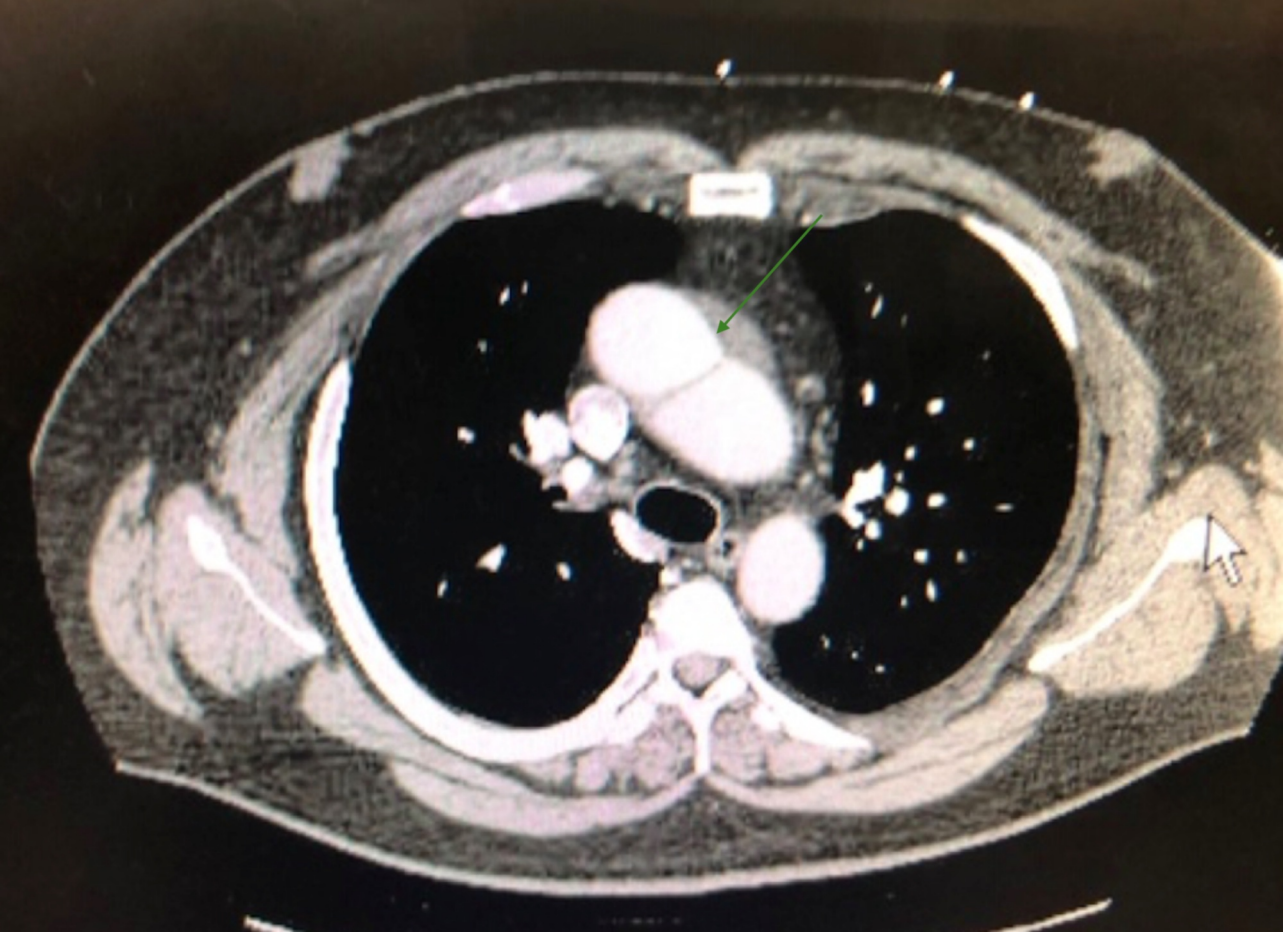
Ascending aorta dissection CT scan of the chest with intravenous contrast showing the aortic dissection.

**Figure 2:**
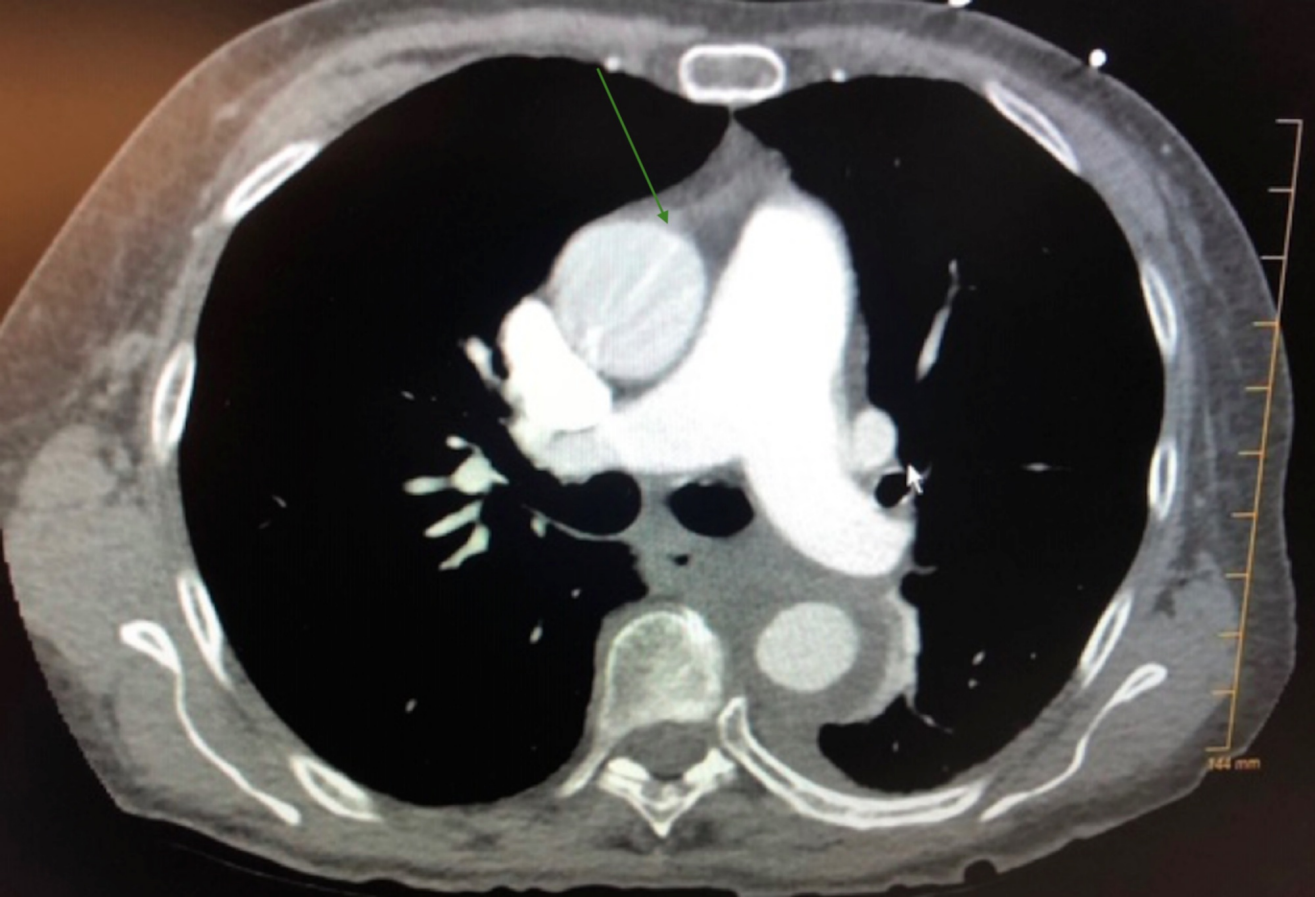
Aortic dissection with extravasation CT scan of the chest with intravenous contrast showing aortic dissection with extravasation.

Since CXR only provides suggestive findings and lacks abdominal visualization, computed tomography angiography (CTA) is the imaging of choice for BTAI, with a reported sensitivity of 98% and specificity of nearly 100% [3]. CTA imaging findings are divided as suggestive and definitive signs for traumatic aortic injury. Suggestive signs are mediastinal or peri-aortic hematoma, retro-crural hematoma, and small caliber of the aorta distal to the injury. Definitive signs are aortic dissection (Figure 1), contained rupture, intramural thrombus, active contrast extravasation (Figure 2), and abnormalities of aortic contour [3]. 

Classifications

Multiple classifications have been proposed over the years (Table 1). Parmley´s classification (1958) is probably the first reported in the literature [6]. This classification is obsolete nowadays because it is pathologically based. Classifications based on CT findings are currently used. For many years, one of the most commonly used classifications was the one proposed by Azizzadeh et al [7]. A modification to this classification was proposed by the Society of Vascular Surgery as intramural hematoma (Grade II) is radiographically poorly defined and can be associated with injuries of adjacent structures and may not represent a true aortic injury [8].

**Table 1:** Traumatic aortic injury classifications

Classification author (year)	Grading	
Parmley's (1958) [6]	Grade 1: Intimal hemorrhage	
	Grade 2: Intimal hemorrhage with a laceration	
	Grade 3: Medical laceration	
	Grade 4: Complete laceration of the aorta	
	Grade 5: False aneurysm formation	
	Grade 6: Periaortic hemorrhage	
Azizzadeh et al. (2009) [7]	Grade 1: Intimal tear	
	Grade 2: Intramural hematoma	
	Grade 3: Aortic pseudoaneurysm	
	Grade 4: Free rupture	
Starnes (2012) [8]	Absent external abnormality	Intimal tear: tear and/or associated thrombus ˂10mm
		Large intimal flap: Tear and/or associated thrombus ˂10mm
	Present external contour abnormality	Pseudoaneurysm: external contour abnormality contained
		Rupture: external contour abnormality not contained, free rupture
Heneghan (2016) "Harborview" [2]	Minimal: no external contour abnormality and an intimal tear or thrombus, or both, sized ˂10mm (Grade 1 - intimal tear, and 2 - intramural hematoma or large intimal flap)	
	Moderate: external contour abnormality or intimal tear ˃10mm. (Grade 3 - pseudoaneurysm)	
	Severe: free rupture, active extravasation.	

The term “minimal aortic injury” (MAI) was coined by Malhotra et al. in 2001 [9]. It was used to describe an intimal flap of less than one centimeter with minimal or no periaortic hematoma. MAI is usually underdiagnosed by CT and diagnosed more accurately with higher-resolution techniques of CTA, intravascular ultrasound (IVUS) or angiography. The term MAI has been used in different studies with varying definitions [3]. Recently, the “Harborview“ classification was proposed by Heneghan et al., which includes the term MAI and guides management based on the grade of injury [2].

Thoracic Aorta

**Figure 3:**
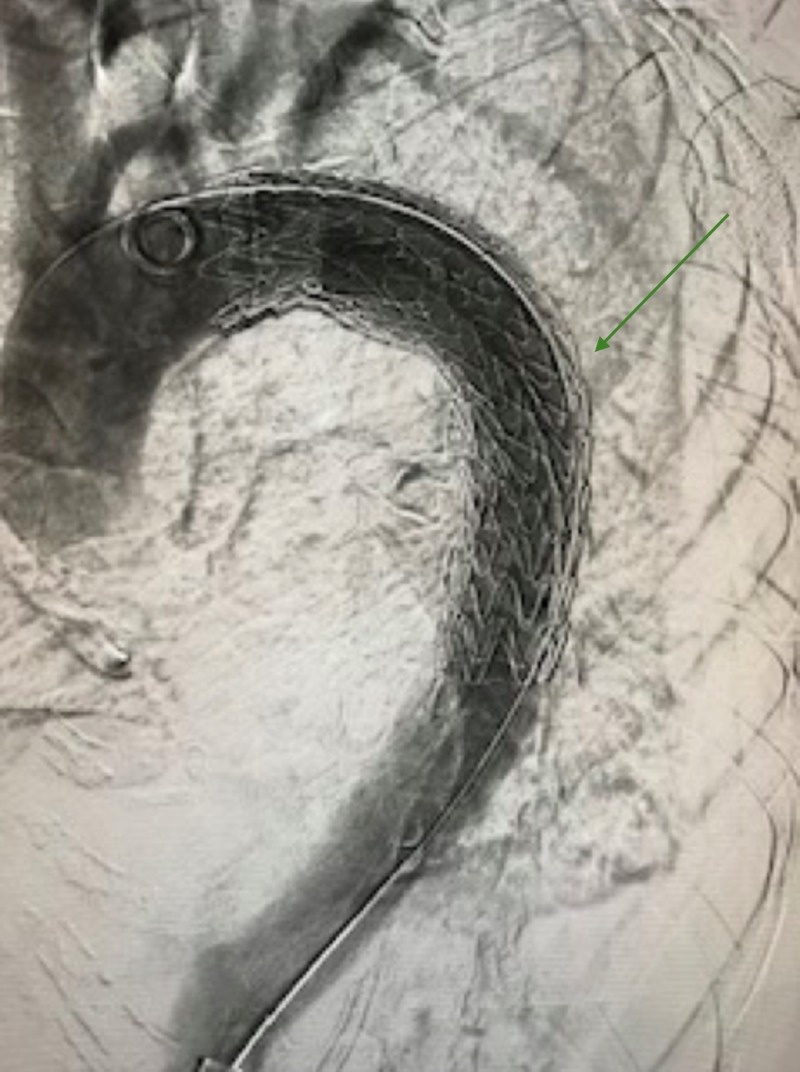
Thoracic endograft

The pre-hospital mortality of BTAI in the thoracic segment is approximately 85%. In-hospital mortality is approximately 19% with open repair and 3% with endovascular repair [10]. According to the Society of Vascular Surgery guidelines from 2011, Grade 1 injuries (intimal tear) warrant expectant management with serial imaging, while Grades 2-4 should undergo repair with thoracic endovascular aortic repair (TEVAR) (Figure 3) [11]. TEVAR has successfully replaced open surgical repair by providing better outcomes, decreasing mortality and minimizing complications associated with open surgical repair (spinal cord ischemia, thoracotomy, aortic cross-clamping and the use of cardiopulmonary bypass) [10, 11].

New treatment strategies for BTAI are leaning towards a more conservative approach. Initial medical management focuses on blood pressure and heart rate control which are the determinants of aortic wall stress, dissection extension and rupture [12, 13]. Targets are usually a heart rate less than 60 bpm and systolic blood pressure between 100-120 mmHg [13]. Beta-blockers are usually the preferred group of agents to control these parameters. For some patients, vasodilators can be required as secondary agents to control blood pressure (Table 2) [12].

**Table 2:** Medical therapy * Alpha and beta receptor antagonist, the advantage of heart rate and blood pressure control from a single agent. ** Viable option for patients with potential contraindication to beta-blockade due to extremely short half-life.

Medica therapy	
Anti-impulse therapy (Beta-blockers)	Propanolol
	Metoprolol
	Labetalol*
	Esmolol**
Vasodilators	Sodium nitroprusside
	Nicardipine
	Nitroglycerin
	Fenoldopam

Delayed aortic endovascular repair (after the first 24 hours) is usually reserved for high-risk patients with associated traumatic injuries. Recently, many studies have shown a decrease in mortality in BTAI stable patients with delayed repair [14]. The Eastern Association for Surgery of Trauma recommends delayed repair with effective blood pressure control in this subset of patients [4]. 

Recommendations for the management of BTAI Grades 1-2 injuries include antiplatelet therapy (aspirin 81 mg for four to six weeks) with no surgical intervention and follow up imaging [2]. Management of Grade 3 (moderate injuries) is based on semi-elective repair during the first 24-72 hours along with optimization of blood pressure and prompt initiation of antiplatelet therapy. Emergency thoracic endovascular aneurysm repair (TEVAR) is reserved for severe (Grade 4) injuries [14]. 

Abdominal Aorta

Abdominal BTAI accounts for only 2.5-5% of blunt aortic trauma [1, 15, 16]. The abdominal aorta extends from the diaphragmatic hiatus to the aortic bifurcation. BTAI is more common at the level of the inferior mesenteric artery (33%) followed by lesions at the level of the renal arteries (24%) [17]. Mortality from blunt abdominal aorta trauma has decreased in the US from 58.3% in 2002 to 26.2% in the last two decades [1].

Although aortic injury classifications are initially based on thoracic injuries, abdominal injuries can be described with the same classification. Intimal flaps <10mm are safely managed with blood pressure control (B-blockers) and antiplatelet therapy (aspirin) [16, 18]. Large intimal flaps, not complicated with thrombosis or arterial insufficiency, are treated with medical management and follow-up CT imaging in 48-72 hours. Large intimal flaps with complications, pseudo-aneurysms, and frank rupture, require urgent endovascular or open surgical repair [16]. Trends are similar to thoracic BTAI management, with an increasing endovascular approach, as an open surgical repair is associated with higher mortality [1, 19]. The use of the endovascular approach for abdominal aortic blunt trauma repair increased from 1.9% in 2002 to 15.9% in 2014 with associated improvement in survival [1].

Blunt traumatic inferior vena cava injury

Blunt traumatic inferior vena cava injury (BTIVCI) occurs only in 1-3.2% of trauma patients [1, 20]. The inferior vena cava (IVC) is divided into infra-renal, para-renal, supra-renal, retro-hepatic, and supra-hepatic. The infra-renal segment is the most commonly injured segment in approximately 39%. The segments with the highest mortality when injured are the retro-hepatic and supra-hepatic [21].

Hospital mortality rates from BTIVCI vary widely, with reports ranging from 49% to 70% [1, 22]. BTIVCI has greater mortality than IVC penetrating injury, abdominal aorta and thoracic aorta [1]. This could be explained by delayed presentation, difficult diagnosis and difficult surgical exposure for repair. Injuries closer to the heart are associated with higher mortality [23]. Hemorrhagic shock, Glasgow Coma Scale and associated injuries are independent factors related to higher mortality rates in BTIVCI [24]. 

BTIVCI mechanisms of injury include violent deceleration shearing forces that can cause a variety of lesions ranging from avulsion of the atrio-caval junction to tearing or intra-parenchymal lacerations [21, 25]. Violent shearing forces contribute to larger and irregular lacerations than the ones observed with IVC penetrating injury [25]. More than half of IVC injuries spontaneously contain the site of injury with the cessation of bleeding by self-tamponade [21]. In stable patients who undergo CT scan, direct signs of IVC injury (such as contrast extravasation) are not easily identified, possibly due to the low caval pressure or tamponade from adjacent structures [22]. However, indirect signs that may help with diagnosis include peri-caval hematoma, irregular caval contour, and filling defects [22, 26, 27].

Management of BTIVCI depends on the hemodynamics of the patient, the location and extent of the lesion and the associated comorbidities. Treatment modalities include definitive vascular repair, damage control procedures (ligation, packing) or conservative approach. The latter usually is restricted to patients who are stable and whose injuries have self-tamponade or contained by adjacent organs (usually the liver, suspensory ligaments, and the diaphragm) [20, 22, 28]. Endovascular therapy for BTIVCI has also up-trended through the years from 0.3% in 2000 to 4.2% in 2014 in the United States. This advance in management is of extreme importance since in-hospital mortality for BTIVCI is most commonly caused by intraoperative exsanguination [21, 29]. BTIVICI with pseudo-aneurysm and/or free rupture has been successfully treated with an endovascular approach [30]. 

The natural history of IVC injuries is not well known and there are fewer reports of follow up compared to aortic injuries. However, conservative management and a combination of endovascular and open repair have been reported in the literature. Cheaito et al. reported a case of a pseudo-aneurysm at the hepatic confluence of the IVC treated conservatively with the total resolution at six months follow-up [20]. Watarida et al. reported the use of a fenestrated stent-graft for blunt traumatic injury of the juxtahepatic IVC with successful control of massive retroperitoneal hematoma with active bleeding [31].

Management with early endovascular management for hemorrhage control, followed by open repair with good results, has also been reported [29].

## Conclusions

Blunt traumatic aortic and IVC injuries remain a leading cause of death worldwide. The complexity of the anatomy and mechanism of injury of these vascular lesions make it difficult to provide a standardized approach. However, the trend in management is shifting towards less aggressive approaches in the proper clinical scenarios. Endovascular management has yielded positive outcomes and correlated with significant improvement in in-hospital survival. A multi-disciplinary team is mandatory for the care of both blunt traumatic aortic and IVC injuries since some cases require hybrid management with endovascular and open surgical repair. Update in guidelines is foreseen in the upcoming years as more techniques are being developed, and more evidence is gathered. Future research will help determine in which cases conservative management provides better outcomes as compared to early interventions. 
